# Use of the Dispersion Coefficient as the Sole Structural Parameter to Model Membrane Chromatography

**DOI:** 10.3390/membranes12070668

**Published:** 2022-06-28

**Authors:** Eleonora Lalli, Giulio C. Sarti, Cristiana Boi

**Affiliations:** Department of Civil, Chemical Environmental and Materials Engineering, DICAM, University of Bologna, Via Terracini 28, 40131 Bologna, Italy; eleonora.lalli@studio.unibo.it (E.L.); giulio.sarti@unibo.it (G.C.S.)

**Keywords:** membrane chromatography, mathematical modelling, dispersion coefficient, dispersivity, pore size distribution

## Abstract

The characterization and modelling of membrane chromatography processes require the axial dispersion coefficient as a relevant and effective intrinsic property of porous media, instead of arbitrary assumptions on pore size distribution. The dispersion coefficient can be easily measured by experiments completely independent of chromatographic tests. The paper presents the prediction of experimentally obtained breakthrough curves using B14-TRZ-Epoxy2 membranes as a test case; the mathematical model implemented is based on the use of the experimentally measured axial dispersion coefficient as an input parameter. Application of the model and its comparison with the data demonstrate that alternative ways of explaining the shape of breakthrough curves, based on unverified assumptions about the membrane pore size distribution, are not feasible and not effectively supported by experimental evidence. In contrast, the axial dispersion coefficient is the only measurable parameter that accounts for all the different contributions to the dispersion phenomenon that occurs in the membrane chromatography process, including the effects due to porous structure and pore size distribution. Therefore, mathematical models that rely on the mere assumption of pore size distribution, regardless of the role of the axial dispersion coefficient, are in fact arbitrary and ultimately misleading.

## 1. Introduction

Membrane chromatography is a relatively new technique that finds relevant application in downstream processing of biological molecules and in biopharmaceutical manufacturing. This technique was originally developed to overcome some of the drawbacks of packed-bed column chromatography [1,2,3], but the limited binding capacity of the first generation of adsorptive membranes has initially prevented its use at the industrial level, except for a few niche applications. With the development of new high-capacity membranes [4,5,6,7], research is needed to bring the technology to full maturity. Efforts to design new modules, studies to make membrane chromatography a continuous process and process intensification all point in this direction [8,9,10,11]. In particular, studying the mass transport of solutes through the porous medium by adopting proper model-based approaches is critical to predicting the performance of such systems, understanding separation mechanisms and optimizing equipment design. To this end, it is important that model parameters are endowed with a clear physical meaning and their value can be obtained from independent direct measurements to enable reliable predictions and scale-up assessments of the systems.

Several mathematical models have been developed to describe transport phenomena and simulate chromatographic processes, most of which originate from the model proposed by Thomas in 1944 [12,13]. Thomas’ model describes heterogeneous ion-exchange chromatography in a packed-bed column for a system with negligible dispersion [12]. This simple model provides an initial estimate of adsorption in chromatographic systems when dispersion is not relevant. Therefore, to better describe adsorption chromatography, a more complete model, such as the general rate model, is needed to account for all mass transfer contributions.

The first mathematical models proposed for membrane adsorption [14,15] are based on mass balance partial differential equations, to be solved simultaneously in time and space. These models also consider extra-column effects in the membrane system, which are relevant when the membrane column volume is of the same order of magnitude as the extra-column volumes. This situation is quite common in laboratory scale preparative systems, where small membrane units, with non-negligible void volumes, are typically applied [16]. Since a comprehensive general rate model is not always necessary for membrane chromatography [17], several simplified forms have been introduced, such as the lumped kinetic model and the lumped pore model [18,19]. These models, however, are generally valid for describing only the system for which the adjustable parameters have been obtained through a best-fitting procedure (improperly referred to as model validation), and they often lose consistency with their physical meaning when systems of different size are considered. For this reason, they cannot be used to effectively describe other experimental systems and are useless for scale-up. Other models are based on empirical approaches, but their use of out of the range of experimental data is not allowed; therefore, they also cannot be used for process scale-up [20]. Of course, mathematical models must be effective and reliable tools for prediction, optimization, and scale-up purposes, they must be as simple as possible and able to effectively describe the main physical phenomena governing the process. They must be based on sound physical and chemical principles, and indeed, there are several models for membrane chromatography based on the fundamental equations of mass transport through porous media that belong to this class of physical models. However, such physical models differ according to the process they describe and the hypothesized mechanisms. In frontal affinity chromatography, there are works based on a transport model that consider pore diffusion, external film resistance, finite kinetics rate and column dispersed flow [21,22]. Different physical models study the effect of input parameters on the shape of the breakthrough curves [23], while other works deal with the accurate study of the adsorption mechanism, describing the interaction between the ligand and the target molecule using the Freundlich adsorption equation [24,25]. A simple and effective physical model based on the use of axial dispersion coefficient for the characterization of porous membranes has been developed and tested by our group with different affinity membranes [26,27,28]. The model is fully predictive, since it requires parameters that represent actual physical properties, whose value can be directly obtained from experiments that are independent of the chromatographic process under investigation.

More recently, a different membrane chromatography model has been proposed that considers pore size distribution as the main factor influencing the separation properties, with the stated aim of adequately accounting for the intrinsic structural properties that influence membrane behavior [29]. This model, in fact, considers only straight cylindrical pores with laminar flow and assumes a suitable pore size distribution, while neglecting other transport phenomena. The pore size distribution is not obtained from separate fluid mechanical tests, nor by direct experimental measurements, but rather is adjusted through a trial-and-error procedure to best fit the observed breakthrough curve in membrane chromatography. This view has also been used to propose improvements for the membrane performance, but actually has the rather unlikely goal of obtaining straight pores with monodisperse diameter distribution.

The purpose of this study is to analyze the effects of dispersion that occur both within the porous medium and in the external ancillary equipment, known as axial dispersion and system dispersion, respectively. It will be shown that all the different contributions to mass transport due to the shape and size distribution of the membrane pores are effectively taken into account by the axial dispersion coefficient, without the need to introduce other detailed structural parameters, such as the pore size distribution itself. Indeed, the axial dispersion coefficient is an intrinsic property of the porous medium. It can be measured experimentally through simple tests independent of the chromatographic process under consideration, such as using injections of concentration pulses, or steps, of tracers under non-binding conditions, and applying the method of moments to the resulting peaks [30,31,32]. Therefore, a simple physical model based on the continuum equation using experimentally accessible transport parameters, such as the dispersion coefficient, without the need to assume any specific pore size distribution is sufficient to obtain and actually predict the breakthrough curves [28]. Such a model is suitable to describe each and every step that contributes to a membrane chromatography process.

In support of the latter claim, we present as a case study membrane affinity chromatography based on non-competitive binding of human IgG on B14-TRZ-Epoxy2 affinity membranes [28,33]. The experimentally obtained breakthrough curves were compared with those predicted by the physical model [28]. The model calculations agree very well with the experimental data under different experimental conditions, thus confirming that the use of the experimental axial dispersion coefficient as an input parameter of the mathematical model, together with the kinetics and equilibrium data, is sufficient to predict the breakthrough curves with very good accuracy, without the need to consider other structural properties of the membrane.

In comparison, the second part of this paper considers the alternative description of membrane behavior based only on assumptions about membrane pore size distribution as suggested by Wei et al. [29], without using any separately measured property as the dispersion coefficient. For a fixed flowrate, one can obtain a pore size distribution that best fits the observed breakthrough curve. However, by considering the effect of flowrate on the breakthrough curves, one realizes that for the same membrane, different flowrates would require different pore size distributions according to the best fit procedure. Equivalently, such physical inconsistency is obtained by calculating the dispersion coefficients for the different pore size distributions associated by the model to the different flowrates, thus leading to the conclusion that the polydisperse membrane assumption offers an artificial and inappropriate membrane chromatography model, with physical inconsistencies and is not applicable for predictions and scale-up purposes.

## 2. Materials and Methods

For the case study, we used B14-TRZ-Epoxy2 affinity membranes that were extensively characterized in batch and dynamic experiments in previous works [28,33,34]. A comparison between experimental data and model prediction is here considered to document that a physical model based on axial dispersion is simple and sufficient for the prediction of breakthrough curves in affinity membrane chromatography, with no need of any assumptions on the membrane pore size distribution.

### Experimental

Bind and elute experiments of human IgG were carried out by performing chromatographic experiments with a fast protein liquid chromatography (FPLC) system, AKTA Purifier 100 (Cytiva, Milan, Italy). All proteins and chemicals were from Merck Life Science srl (Milan, Italy), unless otherwise stated.

Polyclonal human IgG at different concentrations was fed to a layered stack of 5 membranes, total thickness 0.1 cm and cross section area 3.8 cm^2^. The IgG source used is Gammanorm, from Octapharma (Stockholm, Sweden), a polyclonal antibody containing all the four subtypes of human IgG. Phosphate saline buffer 0.1 M, pH 7.4 (PBS) was used in the equilibration and washing stages and to prepare the IgG solutions fed during the loading step. PBS was filtered prior to use, with a 0.45 μm nitrocellulose membrane through a vacuum apparatus, while protein solutions were additionally filtered using a low protein adsorption syringe filter (0.22 μm) purchased from Sartorius Stedim Biotech GmbH (Goettingen, Germany).

Different operating conditions were tested, setting the flowrate at 1, 2, 5, 10 mL/min and changing the IgG concentration in the feed between 0.48 and 2.15 mg/mL.

The moment analysis was carried out on the responses resulting from pulse injections of tracers of different molecular weight, such as acetone, glycine, BSA and IgG, under non-binding conditions; the effluent peaks were analyzed to measure the axial dispersion coefficient.

## 3. Membrane Chromatography Physical Model

The mathematical model used to predict the experimental breakthrough curves was previously developed and validated [28]. In particular, the physical model for membrane chromatography includes transient transport, axial dispersion and kinetics of binding onto the solid surface as the essential phenomena involved in the process, so that

(1) $$\{\begin{array}{c} \varepsilon \frac{\partial c_{i}}{\partial t}+\varepsilon \langle v\rangle \frac{\partial c_{i}}{\partial z}=\varepsilon D_{L}\frac{\partial^{2}c_{i}}{\partial z^{2}}-\left(1-\varepsilon \right)\frac{\partial q_{i}}{\partial t}\\ t=0\;\;\;\;\;\;\;\;\;\;\;\;c_{i}=0 \end{array}$$

In Equation (1), *c_i_* represents the concentration of the species *i* dissolved in the liquid phase, ε the membrane void fraction, *D_L_* the axial dispersion coefficient and *q_i_* is the concentration of adsorbed solute *i* per unit volume of solid the membrane material. If the Langmuir model is adopted to describe the relation between the solute concentration in the solution, *c_i_*, and the concentration of the adsorbed solute on the membrane, *q_i_*, Equation (1) becomes the following equation:

(2) $$\{\begin{array}{c} \frac{\partial c_{i}}{\partial t}\left[1+\frac{\left(1-\varepsilon \right)}{\varepsilon }\frac{q_{m}K_{d}}{{\left(c_{i}+K_{d}\right)}^{2}}\right]+\langle v\rangle \frac{\partial c_{i}}{\partial z}=D_{L}\frac{\partial^{2}c_{i}}{\partial z^{2}}\\ t=0\;\;\;\;\;\;\;\;\;\;\;\;\;\;\;\;\;\;c_{i}=0 \end{array}$$

where *q_m_* and *K_d_* represent the Langmuir adsorption isotherm parameters. In case the bi-Langmuir adsorption model is needed to represent the equilibrium data, the molecules adsorbed on the membrane surface undergo two different types of interactions, due to the existence of two kinds of binding sites [35]; often, one interaction is reversible and one irreversible, thus the isotherm model changes according to Equation (3), which is as follows:

(3) $$q_{i}=q_{i}^{irr}+q_{i}^{rev}=\frac{q_{m}^{irr}c_{i}}{K_{d}^{irr}+c_{i}}+\frac{q_{m}^{rev}c_{i}}{K_{d}^{rev}+c_{i}}$$

Thus, when the bi-Langmuir model is adopted, Equation (1) can be rewritten as follows:

(4) $$\{\begin{array}{c} \frac{\partial c_{i}}{\partial t}\left[1+\frac{\left(1-\varepsilon \right)}{\varepsilon }\left(\frac{q_{m}^{irr}K_{d}^{irr}}{{\left(c_{i}+K_{d}^{irr}\right)}^{2}}+\frac{q_{m}^{rev}K_{d}^{rev}}{{\left(c_{i}+K_{d}^{rev}\right)}^{2}}\right)\right]+\langle v\rangle \frac{\partial c_{i}}{\partial z}=D_{L}\frac{\partial^{2}c_{i}}{\partial z^{2}}\\ t=0\;\;\;\;\;\;\;\;\;\;\;\;c_{i}=0 \end{array}$$

The void fraction and axial dispersion coefficient can be measured directly by applying the moment analysis method to the effluent peaks resulting from the pulse injection tests, under non-binding conditions. According to the moment analysis, in the case of packed column, the following relationships hold true for the first moment, $\mu_{1}$, and the second central moment, ${\bar{\mu }}_{2}$ [28]:

(5) $$\mu_{1}=\frac{M_{1}}{M_{0}}\equiv \frac{\int_{0}^{\infty }f(t)tdt}{\int_{0}^{\infty }f(t)dt}=\frac{L_{}}{\langle v\rangle }$$

(6) $${\bar{\mu }}_{2}\equiv \frac{\int_{0}^{\infty }f(t){\left(t-\mu_{1}\right)}^{2}dt}{\int_{0}^{\infty }f(t)dt}=2L_{}\frac{D_{L}}{\varepsilon {\langle v\rangle }^{3}}$$

where *L* represents the total thickness of the membrane stack and 〈v〉 the average interstitial velocity.

When applied to the effluent peaks observed in non-binding conditions, Equation (6) allows one to directly calculate the axial dispersion coefficient in a straightforward way.

The adsorption equilibrium conditions can be measured directly, e.g., in batch mode, thus obtaining the information on which the adsorption isotherm is appropriate and what its parameter values are. Many affinity chromatography systems are well described by the Langmuir isotherm, although in some cases, Freundlich, Temkin and bi-Langmuir might be more appropriate [14,15,24,36,37]. In the case under consideration, the bi-Langmuir isotherm was found appropriate, with the parameter values reported in Table 1.

Aspen Custom Modeler^TM^ was used to implement Equation (4) and to obtain the predicted breakthrough curves, using the experimental operative conditions and the measured axial dispersion coefficient as input data. All information related to external column volumes, equilibrium parameters, as well as the value of the axial dispersion coefficient, are listed in Table 1.

### Physical Model Simulations of Breakthrough Curves

**Figure 1:**
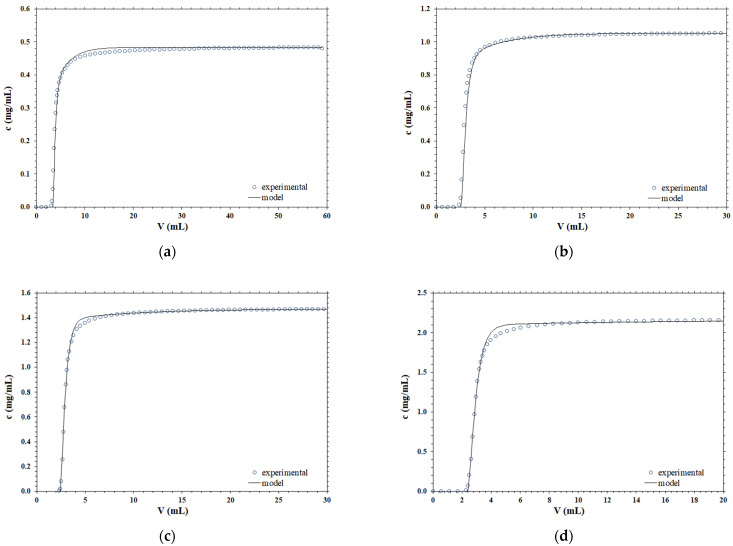
Comparison between the experimental and simulated breakthrough curves at different operating conditions; (**a**) *F* = 1 mL/min, *c*_0_ = 0.48 mg/mL; (**b**) *F* = 2 mL/min, *c*_0_ = 1.05 mg/mL; (**c**) *F* = 5 mL/min, *c*_0_ = 1.47 mg/mL; (**d**) *F* = 10 mL/min, *c*_0_ = 2.15 mg/mL.

Different experimental breakthrough curves, obtained for B14-TRZ-Epoxy2 membranes using various operating conditions, are compared with the corresponding predicted curves using the mathematical model embodied by Equation (4). The results obtained are presented in Figure 1.

According to the data presented, it is clear that the shape of the breakthrough curve is very well described by the dispersion term, at all the concentrations and flowrates considered, by using parameter values that are obtained from separate independent experimental measurements and are not the result of a best fitting to the chromatography breakthrough curves.

## 4. Polydispersed Membrane Theory

In order to properly compare the capabilities of the two different approaches, we consider now the alternative model proposed by Wei et al. that associates the shape of breakthrough curves to the pore size distribution of a hypothetical polydispersed membrane [29], and apply it to the same experimental data of the case study examined.

Of course, general mathematical models suitable for membrane chromatography must describe well the relevant kinetic and transport phenomena occurring in all different stages of the chromatographic cycle. However, to perform a meaningful comparison between the two approaches and their effectiveness, it is sufficient to consider only the adsorption stage with negligible binding kinetics. Therefore, washing and elution stages do not add any interesting information to our aim and will be disregarded. Similarly, the attention will be focused on the chromatographic module only and all the effects of fluid dynamics in the external volumes of the chromatographic system are not taken into consideration.

Breakthrough curves will be obtained for a polydispersed membrane model according to its assumptions, and will be adjusted to experimental data. The corresponding dispersion coefficient will be calculated, using the method of moments, when a solution of a generic solute *i* flows through a membrane column under non-binding conditions. Finally, the results of the polydispersed membrane model will be compared to those of the physical model, as described in § 3, which uses the axial dispersion coefficient as the input parameter instead of the pore size distribution to point out the different model capabilities.

### 4.1. Membrane Column Model

The membrane column is considered as an ideal porous medium with uniform porosity; in particular, the module is composed of one membrane disc of known diameter. The features of the membrane, in terms of thickness, void fraction and average pore size, refer to commercial membranes of the Sartobind^®^ family (Sartorius Stedim Biotech GmbH); the relevant data, according to the data sheet, are reported in Table 2.

According to the model by Wei et al. [29], the membrane pores are schematized as parallel cylinders of radius *r_p_* whose average equals the average pore radius and with length equal to the membrane thickness. The pore size distribution of the membrane, *f*(*r_p_*), can be approximated by the Gaussian expression given in Equation (7), consistent with the fact that the measured pore size distribution of commercial membranes does not differ much from a Gaussian type. The quantity $f\left(r_{p}\right)dr_{p}$ represents the fraction of pores whose diameter is between *r_p_* and *r_p_* + *dr_p_*.

**Figure 2:**
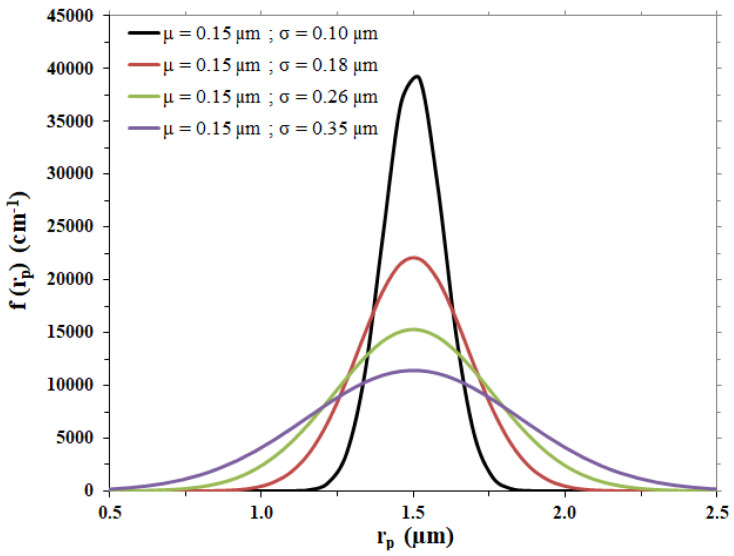
Gaussian pore size distributions hypothesized for the membrane disc.

For the case under examination, different Gaussian curves were considered to represent possible membrane pore size distributions, with the same average pore radius, µ, as the one of the membrane considered and different standard deviation values, σ; calculations were performed with the values listed in Table 3, leading to the distribution trends shown in Figure 2.

(7) $$f(r_{p})=\frac{1}{\sqrt{2\pi }\sigma }\exp\left[-\frac{1}{2}{\left(\frac{r_{p}-\mu }{\sigma }\right)}^{2}\right]$$

The interstitial flow velocity, i.e., the ratio between volume flowrate and the overall cross-sectional area of the void channels, is assumed to be constant and radially uniform in the membrane column, with no velocity component in the radial direction. Therefore, there is no variation in the concentration of all the species along the membrane radius.

The model takes into account only axial convection and longitudinal dispersion, which is due to the following two simplifying assumptions [29]: (i) the contribution of molecular diffusion to the motion of the generic solute *i* is negligible; (ii) the flow velocity of the solution inside each pore is considered constant over the cross section of the pores themselves and equal to the average velocity in the pore. The last assumption implies, in particular, that the Taylor–Aris dispersion is neglected.

### 4.2. Model Equations

According to the assumptions on the pore structure formed by parallel cylindrical pores endowed with laminar flow, and considering the pore size distribution *f(r_p_)*, one can first calculate the volume flowrate *F(r_p_)* and average velocity *v_m_(r_p_)* associated to each pore of the membrane. Then, one can relate the time evolution of the concentration exiting each pore to the time dependence of the inlet concentration, and finally, one can obtain the time evolution of the average concentration at the membrane exit that represents the breakthrough curve.

The total number of pores *n_p_* is obtained from the overall cross-sectional area using the following equation:

(8) $$n_{p}=\frac{\varepsilon A_{m}}{\pi \int_{0}^{+\infty }r_{p}^{2}\;f\left(r_{p}\right)dr_{p}}$$

For the volume flowrate *F(r_p_)* through the pore of radius *r_p_* and for the overall volume flowrate *F,* one can use the following equation:

(9) $$F\left(r_{p}\right)=\frac{\Delta P}{8\eta L}\pi r_{p}^{4}$$

(10) $$F=\frac{\Delta P}{8\eta L}\overset{+\infty }{\underset{0}{\int }}\pi r_{p}^{4}\;n_{p}f\left(r_{p}\right)dr_{p}$$

where Δ*P* is the overall pressure difference and *η* is the liquid viscosity. In view of Equations (8)–(10) one has the following equation:

(11) $$F\left(r_{p}\right)=\frac{F}{n_{p}}\frac{r_{p}^{4}}{\int_{0}^{+\infty }r_{p}^{4}\;f\left(r_{p}\right)dr_{p}}=\frac{F}{\varepsilon A_{m}}\;\frac{\pi r_{p}^{4}\int_{0}^{+\infty }r_{p}^{2}\;f\left(r_{p}\right)dr_{p}}{\int_{0}^{+\infty }r_{p}^{4}\;f\left(r_{p}\right)dr_{p}}$$

Therefore, the average velocity in the pore of radius *r_p_* is obtained as follows:

(12) $$v_{m}\left(r_{p}\right)=\frac{F}{\varepsilon A_{m}}\frac{r_{p}^{2}\int_{0}^{+\infty }r_{p}^{2}\;f\left(r_{p}\right)dr_{p}}{\int_{0}^{+\infty }r_{p}^{4}\;f\left(r_{p}\right)dr_{p}}\equiv \langle v\rangle \frac{\int_{0}^{+\infty }r_{p}^{2}\;f\left(r_{p}\right)dr_{p}}{\int_{0}^{+\infty }r_{p}^{4}\;f\left(r_{p}\right)dr_{p}}\;r_{p}^{2}$$

The residence time *τ(r_p_)* in the pore of radius *r_p_* is given by $\tau (r_{p})=L/v_{m}(r_{p})$, so that under non-binding conditions, the concentration exiting the pore of radius *r_p_* is immediately obtained as follows:

(13) $$c_{i}^{out}(t,r_{p})=c_{i}^{in}\left(t-\tau (r_{p}),r_{p}\right)\equiv c_{i}^{in}\left(t-\tau (r_{p})\right)$$

The second equality in Equation (13) is due to the fact that the inlet concentration is the same for all the pores. Finally, from the total molar flowrate exiting the membrane, one obtains the time dependence of the average concentration exiting the membrane. In view of Equation (11) one immediately has the following equation:

(14) $$c_{i,ave}^{out}\left(t\right)=\frac{1}{F}\overset{+\infty }{\underset{0}{\int }}F\left(r_{p}\right)c_{i}^{out}\left(t,r_{p}\right)\;n_{p}f\left(r_{p}\right)dr_{p}=\frac{\int_{0}^{+\infty }r^{4}c_{i}^{in}\left(t-\tau \left(r_{p}\right)\right)\;f\left(r_{p}\right)dr_{p}}{\int_{0}^{+\infty }r^{4}\;f\left(r_{p}\right)dr_{p}}$$

which embodies the breakthrough curve.

The obtained breakthrough curve derives from a finite discretization of the pore radius and it has to be regularized by fitting the data with an appropriate relation. A sigmoid function properly modified, Equation (15), was found appropriate and was chosen to fit and smoothen the breakthrough curve.

(15) $$\{\begin{array}{l} t\leq t_{lag},\;c_{i,out}=0\\ t>t_{lag},\;c_{i,out}\equiv c_{i,ave}^{out}\left(t\right)=\frac{a}{1+\exp\left\{-b\left[\left(t-t_{lag}\right)-c\right]\right\}}-\frac{a}{1+\exp(b\;c)} \end{array}$$

The fitting equation has three adjustable parameters, *a*, *b* and *c*, plus the quantity *t_lag_*, that represents the time up to which the concentration exiting the membrane module is equal to zero.

Since the aim of the proposed model is the determination of the axial dispersion coefficient that can be easily obtained by applying the method of moments [16,17,22], the response of the membrane column to a concentration pulse has to be studied. In this regard, it is useful to be reminded that the response to a pulse is a peak that can be obtained as the derivative of the breakthrough curve.

(16) $$\{\begin{array}{l} t\leq t_{lag},\;\frac{dc_{i,out}}{dt}=0\\ t>t_{lag},\;\frac{dc_{i,out}}{dt}=\frac{a\;b\;\exp\left\{-b\left[\left(t-t_{lag}\right)-c\right]\right\}}{{\left\{1+\exp\left\{-b\left[\left(t-t_{lag}\right)-c\right]\right\}\right\}}^{2}} \end{array}$$

By applying Equation (8) to the peaks resulting from Equation (16) the axial dispersion coefficient can be easily obtained for each pore size distribution. Microsoft Excel^®^ was used to implement Equations (8)–(16), which were solved to obtain the breakthrough curves and the corresponding axial dispersion coefficient.

#### Model Validation

The path followed to validate the simple model proposed is a theoretical comparison with a rigorous mathematical model, which was previously developed and validated in our research group [28], expressed by Equation (1). The assumptions made for the development of the simple model focus on the breakthrough curve under non-binding conditions, and thus reduce the physical model governing equation, Equation (13), to the following:

(17) $$\left\{ \begin{array}{l} \frac{\partial c_{i,out}}{\partial t}+\langle v\rangle \frac{\partial c_{i,out}}{\partial z}=D_{L}\frac{\partial^{2}c_{i,out}}{\partial z^{2}}\\ t=0\begin{array}{cc} & c_{i}=0 \end{array} \end{array}\right.$$

**Figure 3:**
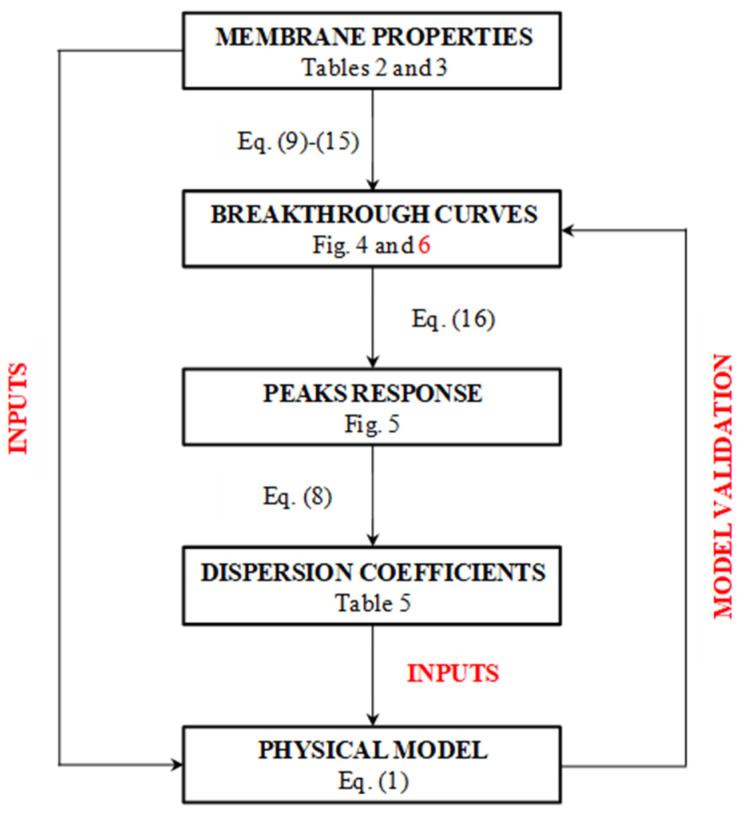
Block diagram of the logical path used to validate the model with references to equations and tables in the text.

This partial differential equation was solved using Aspen Custom Modeler^TM^, imposing the same input data used for the simplified model. The dispersion coefficient calculated with the simplified model was also used as an input for the physical model, whose resulting breakthrough curves were compared to those obtained with the simplified model, as a demonstration of the hypothesis made at the beginning. Figure 3 shows a schematic representation of the method followed to validate the proposed model.

## 5. Results and Discussion

**Figure 4:**
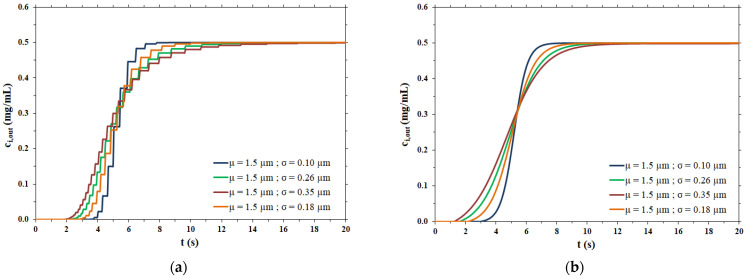
(**a**) Breakthrough curves as a function of the pore size distribution; (**b**) fitting of breakthrough curves as function of the pore size distribution.

The breakthrough curves obtained at a constant flowrate of 1 mL/min considering a membrane disc of 2.2 cm diameter for the hypothetical polydispersed membranes are shown in Figure 4.

As expected, the breakthrough curves are steeper for membranes with a narrow pore size distribution, corresponding to a low value of the standard deviation σ, and broaden as the pore size distribution broadens.

**Figure 5:**
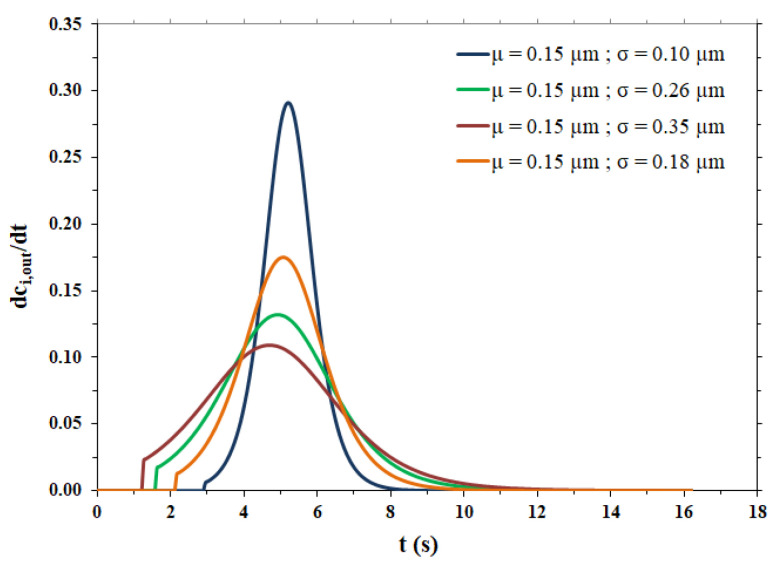
Pulse response peaks for the different pore size distributions.

The fitting parameters and the lag time related to the breakthrough curves shown in Figure 4b are reported in Table 4, while the pulse response peaks derived from the same curves of Figure 4b are shown in Figure 5.

As expected, the more dispersed the pore size distribution, the more the output peaks are broad, tailed and, consequently, less high. All peaks show irregular behavior for times near *t_lag_*, particularly the peaks related to broad pore size distributions. The steps are due to the range of variation in the pore radius, as shown in Table 3. In particular, the maximum value of the pore radius is responsible for an abrupt change in the flow exiting the membrane (breakthrough curve) and this causes an even more abrupt change in the impulse response peak (derivative of the breakthrough curve). However, it was calculated that if the maximum value of the range of variation in the pore radius is increased from 2.5 × 10^−4^ cm to 6.5 × 10^−4^ cm, to limit the irregular behavior near *t_lag_*, the dispersion coefficient undergoes a change of only 3.16% in the case of the broadest pore size distribution (highest σ value) and the breakthrough curve remains practically the same.

Applying the method of moments to these curves and, in particular, taking advantage of the second central moment in Equation (6), the corresponding dispersion coefficients were calculated and summarized in Table 5.

**Figure 6:**
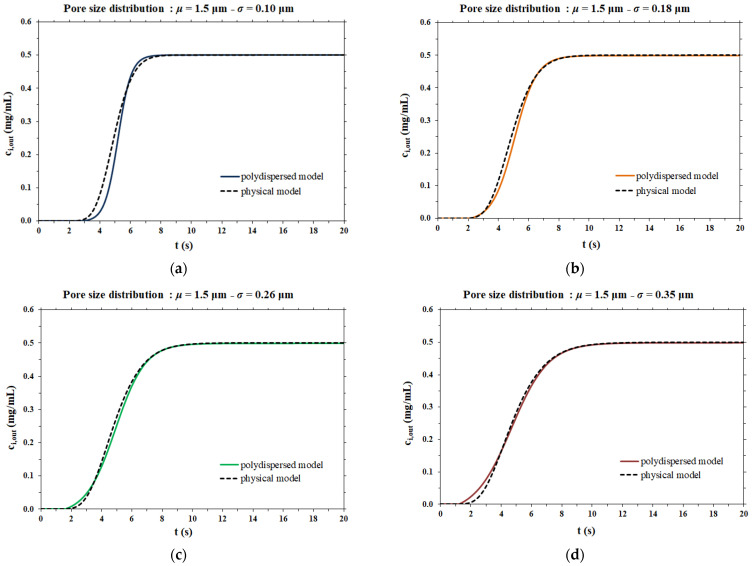
Comparison between the model proposed in this work and the physical model for different pore size distributions; (**a**) *µ* = 1.5 µm and *σ* = 0.10 µm; (**b**) *µ* = 1.5 µm and *σ* = 0.18 µm; (**c**) *µ* = 1.5 µm and *σ* = 0.26 µm; (**d**) *µ* = 1.5 µm and *σ* = 0.35 µm.

The dispersion coefficient values thus obtained were used as the input data for the rigorous physical model of membrane chromatography. The breakthrough curves computed by the two mathematical models considered are compared side by side in Figure 6.

In the case of a narrow pore size distribution (Figure 6a), the contribution to dispersion is mainly from the velocity distribution (resulting in the parabolic profile of flow velocity within each pore), meaning that the Taylor–Aris dispersion is the dominant phenomenon. On the other hand, when the pore size distribution is broad (Figure 6d), the contribution of Taylor–Aris to the overall dispersion phenomenon is less relevant, while the pore size distribution represents the main contribution to dispersion. For this reason, the results of the two models agree more in the case of a broad pore size distribution than in the case of a narrow pore size distribution.

In all cases, the two models agree very well at all stages of breakthrough, indicating that the physical model approach that makes use of a dispersion coefficient is a more efficient way to describe the membrane chromatographic process. Indeed, it is based on the use of a macroscopic physical property of the porous medium that can be measured independently, and not on an arbitrary assumption on the porous structure. In addition, the hypothesis of the polydispersed membrane model to describe the shape of breakthrough curves is not only unnecessarily cumbersome and time consuming, but more importantly, has inconsistencies with the actual physical response observed in the membrane chromatography process. This can be easily demonstrated by considering the effect of flowrate on the breakthrough curve resulting from a feed pulse of a non-binding tracer. As noted earlier, these curves allow one to obtain the dispersion coefficient of the porous medium, *D_L_*, as well as the dispersivity coefficient, *α*, which is defined as

(18) $$\alpha =D_{L}/\langle v\rangle$$

The dispersivity coefficient *α* has the advantage of being independent of the average velocity used 〈v〉 [18,19], and is, therefore, an effective property of the material, as is the actual pore size distribution.

The polydisperse membrane model was used to calculate the breakthrough curves from a pulse of a non-binding species, considering three different pore size distributions (same average diameter, different standard deviation) and four different flowrates for each. The corresponding dispersion coefficients *D_L_* and dispersivity coefficients *α* were calculated and reported in Table 6. Clearly, for each pore size distribution, the values of the dispersivity coefficient, α, vary with the flowrate, which is conceptually wrong, since α is a physical parameter of the membrane. The actual dispersivity coefficient should be constant for each membrane, and thus have a single value for each pore distribution under investigation.

## 6. Conclusions

The dispersion coefficient is an important parameter for modelling membrane chromatography processes. It is intrinsically linked to the porous nature of the stationary phase used for this separation technique and it can be easily measured through independent experimental tests, such as pulse or step injections of tracer molecules, under non-binding conditions, and using the method of moments. The key role of this parameter relies on the fact that it encloses all different contributions to axial dispersion, without the need of arbitrary unverified assumptions on shape and size distribution of the pores, and on the flow velocity profile in each pore. It has been shown that for a given flowrate, the breakthrough curve derived by hypothesizing a polydispersed membrane may be the same breakthrough curve obtained by applying a physical model, which uses the experimentally measured axial dispersion coefficient as an input parameter. However, the polydispersed membrane model proposed in ref. [29] leads to unacceptable inconsistencies by considering the effect on the breakthrough curves of different flowrates. Indeed, it provides a dispersivity coefficient that varies with flowrate for a given membrane, contrary to the fact that the dispersivity coefficient is a material property and has a single value, independent of average velocity, for any given membrane.

The case study presented here shows that experimental binding data for membrane chromatographic systems are very well reproduced using the physical model, without the need to consider the membrane pore size distribution, which, in the published works, is not separately measured, but improperly obtained as a result of fitting the experimental breakthrough data.

This work demonstrates that to effectively model membrane chromatography, it is essential to use the membrane dispersion coefficient as an input parameter to obtain results consistent with the physics of the process, suitable for predictions and scale-up purposes.

## Figures and Tables

**Table 1:** Geometrical constants, system volumes and bi-Langmuir parameters related to the experimental system [28,33].

Geometrical Data		Dispersion		Isotherm Parameters		
*L* (cm)	0.1 ^a^	*V_CSTR_* (mL)	0.69		Irreversible binding	Reversible binding
*A* (cm^2^)	3.8	*V_PFR_* (mL)	0.025∙*F* +1.753 ^b^	*K_d_* (mg/mL)	0	1.15
*ε*	0.545	*α* (cm)	0.104 ^c^	*q_m_* (mg/mL)	4.75	7.00

^a^ Total length for a stack of 5 membranes. ^b^ The PFR volume was calculated according to the flowrate, F. ^c^ Dispersivity coefficient; $\alpha =D_{L}/\langle v\rangle$.

**Table 2:** Sartobind^®^ Q membrane specifications [38].

Pore Radius	Void fraction	Thickness
(μm)	(%)	(μm)
1.50	80	275

**Table 3:** Pore size distribution parameters.

*μ*	*σ*	Minimum Pore Radius	Maximum Pore Radius
(μm)	(μm)	(μm)	(μm)
1.50	0.10; 0.18; 0.26; 0.35	0.50	6.50

**Table 4:** Fitting parameters of the breakthrough curves in Figure 4b.

Pore Size Distribution	Fitting Parameters			*t_lag_*
*µ* = 0.15 µm *σ* = 0.10 µm	a=0.50	b=2.32	c=2.26	$t_{lag}=2.94\;s$
*µ* = 0.15 µm *σ* = 0.18 µm	a=0.51	b=1.38	c=2.90	$t_{lag}=2.16\;s$
*µ* = 0.15 µm *σ* = 0.26 µm	a=0.52	b=1.02	c=3.29	$t_{lag}=1.62\;s$
*µ* = 0.15 µm *σ* = 0.35 µm	a=0.53	b=0.83	c=3.43	$t_{lag}=1.26\;s$

**Table 5:** Dispersion coefficient for each pore size distribution.

Pore Size Distribution		Dispersion Coefficient
*µ* (µm)	*σ* (µm)	*D_L_* (cm^2^/s)
1.5	0.10	$1.38\times 10^{-6}$
1.5	0.18	$3.63\times 10^{-6}$
1.5	0.26	$6.17\times 10^{-6}$
1.5	0.35	$8.77\times 10^{-6}$

**Table 6:** Values of the dispersion and dispersivity coefficients with flowrate calculated with the polydispersed model.

Pore size Distribution Parameters		*F* = 10 mL/min		*F* = 5 mL/min		*F* = 2 mL/min		*F* = 1 mL/min	
*μ* (µm)	*σ* (µm)	*D_L_* (cm^2^/s)	*α* (cm)	*D_L_* (cm^2^/s)	*α* (cm)	*D_L_* (cm^2^/s)	*α* (cm)	*D_L_* (cm^2^/s)	*α* (cm)
1.5	0.10	1.69 × 10^−5^	3.089 × 10^−4^	7.81 × 10^−4^	2.850 × 10^−4^	2.96 × 10^−6^	2.704 × 10^−4^	1.38 × 10^−6^	2.518 × 10^−4^
1.5	0.26	7.70 × 10^−5^	1.404 × 10^−3^	3.70 × 10^−5^	1.350 × 10^−3^	1.44 × 10^−5^	1.314 × 10^−3^	6.17 × 10^−6^	1.125 × 10^−3^
1.5	0.35	1.04 × 10^−4^	1.892 × 10^−3^	5.24 × 10^−5^	1.911 × 10^−3^	2.13 × 10^−5^	1.941 × 10^−3^	8.77 × 10^−6^	1.600 × 10^−3^

## Data Availability

The data presented in this study are available on request from the corresponding author.
